# Increasing trend of scabies in Belgium, 2000–2023

**DOI:** 10.1186/s13690-025-01684-3

**Published:** 2025-07-29

**Authors:** Valeska Laisnez, Isabel Brosius, Wim Van Bortel, Marie Meudec, Arne Janssens, Julia Madl, Amba Josiane Aye, Lien Bruggeman, Lode Godderis, Wouter Dhaeze, Muriel van Durme, David Hercot, Ive Talboom, Julie Bossu, Blaise Barche, Lucy Catteau, Soledad Colombe

**Affiliations:** 1 Sciensano, Brussels, Belgium; 2 ECDC Fellowship Programme, Field Epidemiology path (EPIET), European Centre for Disease Prevention and Control (ECDC), Stockholm, Sweden; 3 Institute of Tropical Medicine, Antwerp, Belgium; 4 KU Leuven, Leuven, Belgium; 5 Médecins du Monde Belgique, Brussels, Belgium; 6 Fedasil, Brussels, Belgium; 7 IDEWE, External Service for Prevention and Protection at Work, Heverlee, Belgium; 8 Department of Care Flemish Region, Brussels, Belgium; 9 AVIQ, Charleroi, Belgium; 10 Vivalis, Brussels, Belgium; 11 Studentengezondheidscentrum KU Leuven, Leuven, Belgium; 12 Studentenartsen UGent, Ghent, Belgium; 13 Université de Mons, Mons, Belgium

**Keywords:** Scabies, *Sarcoptes scabiei*, Skin diseases, Mite infestations, Belgium

## Abstract

**Background:**

Several European countries have reported an increasing trend of human scabies in the past twenty years. In Belgium, scabies’ clusters hinted at a similar trend despite individual cases not being notifiable. We aimed to describe scabies’ trends between 2000 and 2023 in Belgium.

**Methods:**

We described the evolution of scabies during 2000–2023 in Belgium using a variety of available data sources. Using generalized linear models, we analysed yearly trends and seasonality of scabies diagnoses at general practitioners (GPs) in Flanders, occupational health services in Belgium, nationwide asylum seekers’ shelters and consultations for people with precarious housing, and student medical facilities in Flanders. Additionally, we analysed national reimbursement and sales data for first-line scabies treatments in Belgium.

**Results:**

Scabies diagnoses significantly increased at all investigated levels (*p*-value < 0.001): primary care (15% yearly increase, 2011–2023), occupational health services (16% yearly increase, 2011–2022, temporary decrease in 2020–2021), asylum seekers’ shelters (41% yearly increase, 2016–2022), consultations for people in precarious living situations (41% yearly increase, 2019–2022), and pharmaceutical sales (15% yearly increase in permethrin sales, 2012–2022). In primary care, those 15–24 years-old were most affected (13.2/1,000 compared to 4.1/1,000 for all ages in 2023), as were urban areas (6.7/1,000 compared to 2.7/1,000 in rural areas in 2022). Incidence was overall higher in males (*p*-value 0.016).

**Conclusions:**

Our study shows a strong increasing trend of scabies in Belgium since 2011, with higher incidences in younger age groups, urban areas and during colder months. We recommend further studies to better quantify the increase and investigate the underlying drivers.

**Table:** 

Text box 1. Contributions to the literature	
• Provides the first multi-source analysis of scabies trends in Belgium (2000–2023), addressing a major surveillance gap. • Confirms a marked and sustained rise in scabies cases, reflecting similar trends reported in other European countries. • Identifies high-risk groups, including young adults, males, and urban residents. • Demonstrates the value of alternative data sources—such as pharmaceutical sales—for surveillance in settings lacking mandatory reporting. • Supports the need for targeted and coordinated public health responses and future research into drivers of scabies.	

## Background

Human scabies is a skin infestation caused by the mite *Sarcoptes scabiei var. hominis*, leading to intense itching and rash [1]. Transmission occurs mainly through prolonged skin-to-skin contact and, less frequently, indirectly via infested items (such as bedding or clothing). Scabies can cause significant discomfort to the patient, with severe nocturnal pruritus. Additionally, those affected often face stigmatization due to the misconception that scabies is linked to poor hygiene [2]. Complications such as bacterial skin infections sometimes followed by acute post-streptococcal glomerulonephritis and rheumatic fever or rheumatic heart disease further add to the burden of disease. Several treatment options are available, both topical and oral, all with a number of limitations. Topical treatments have to be applied to the entire body while oral treatment is often costly. Close contacts also have to be treated and extensive hygienic measures are required to eliminate the mites and prevent further transmission.

Since 2017, the World Health Organization has recognized scabies as a neglected tropical disease. According to 2023 estimates, scabies affects more than 400 million people each year worldwide [3]. Though historically considered as a resource-poor and tropical-settings’ disease, several European countries have reported a striking increase in scabies infestations over the last two decades, especially among adolescents and young adults [4–11]. Potential drivers for this trend include increased drug resistance, diagnostic delays or misdiagnosis, burden and cost of treatment, high social connectivity and mobility in young adults as well as increased travel and migration [4–9].

In recent years there were also signs of an increase in scabies in Belgium [12, 13]. Through informal discussions, it was reported that general practitioners (GPs) and dermatologists had been seeing an overall increasing number of patients and pharmacists had been receiving more questions from patients about scabies. A higher number of clusters were also reported to the Regional Health Authorities (RHAs), especially in settings where response is more challenging due to structural barriers or complex household situations (e.g. blended families). Individual cases of scabies are not reportable by law in Belgium, only clusters of scabies infection (two or more cases) occurring in community (non-family) settings must be reported. This makes monitoring of trends and identification of risk factors difficult. Therefore, this study aimed to map the available data sources related to scabies in Belgium and to describe the evolution of scabies during 2000–2023, to better inform public health stakeholders and to design more targeted research studies.

## Methods

### Setting/study site

Belgium is divided into three regions: Flanders, Wallonia and Brussels, each with its own RHA. Scabies is diagnosed by GPs and dermatologists. Diagnosis of scabies is typically based on clinical evaluation including anamnesis and a subset of cases is confirmed by microscopy on skin scrapings or by dermatoscopy [14]. There is no standard follow-up of patients and no confirmation of cure. The Belgian Antibiotic Policy Coordination Commission (BAPCOC), advices the use of permethrin (topical application) as first-line treatment in outpatient practice, with ivermectin (oral or topical) and benzyl benzoate (topical) as alternatives [15]. Permethrin is reimbursed since November 2019 in Belgium. Oral ivermectin has been available only since 2022 for treating scabies (previously off-label use) but was not reimbursed until June 2025 [16]. Topical benzyl benzoate has been reimbursed since before 2000.

### Study design

We conducted a descriptive, retrospective study of scabies diagnoses and treatment records in Belgium from 2000 until 2023.

### Data collection

As summarized in Table 1, non-mutually exclusive data from 11 national and regional sources were explored and triangulated to describe trends in scabies in Belgium, including diagnoses of scabies at general medical practices, occupational health services, specific student health services, shelters for asylum seekers and medical consultations for people in precarious housing situation, as well as data on treatment reimbursement and sales. The data from these different sources were only available for certain time periods and these could not be homogenized. We considered diagnoses corresponding to the code S72 (‘scabies/other acariasis’) of the International Classification of Primary Care, second edition (ICPC2) as scabies (abbreviated as ‘scabies diagnoses’ throughout the rest of this manuscript) [17].

**Table 1:** Data sources with target group, available time period and interval, and demographic information

Reference	Source	Target group	Time period and interval	Information available	Geographical level	Age interval	Sex
1	INTEGO public tool	General population	2000–2023, yearly	Number of ICPC2 S72 codes per yearly contact group	Regional (Flanders)	5 years	M/F
2	INTEGO research data	General population	2011–2016, quarterly	Number of ICPC2 S72 codes per yearly contact group	Regional (Flanders)	5 years	M/F
3	INTEGO research data	General population	2017–2022, quarterly	Number of ICPC2 S72 codes per yearly contact group	City (Antwerp, Ghent, other urban and rural in Flanders)	5 years	M/F
4	IDEWE	Employees	2011–2022, yearly	Number of self-reported scabies diagnoses	National service but more active in Flanders	NA	NA
5	Fedasil	Asylum seekers	2016–2022, yearly	Number of scabies diagnoses in inhabitants of refugee centers, over mean number of occupants in the centers during that year	National	NA	NA
6	Doctors of the World	People in precarious living situations	2019–2022, yearly	Number of ICPC2 S72 codes per number of consultations	National	5 years	M/F/O
7	Student medical services Leuven	Students	2013–2022, yearly	Number of scabies diagnoses over number of consultations	City (Leuven)	NA	NA
8	Student medical services Ghent	Students	2020–2022, yearly	Number of scabies diagnoses over number of consultations	City (Ghent)	NA	NA
9	IQVIA	General population	2012–2022, yearly	Number of packages of topical permethrin (Zalvor^®^) sold by wholesalers to Belgian community pharmacies	National	NA	NA
10	NIHDI	General population	Nov 2019-March 2023, monthly	Number of packages of topical permethrin (Zalvor^®^) reimbursed in Belgium	Province	0–19 20–59 ≥ 60	M/F
11	APB	General population	Jan 2019-February 2023, monthly	Number of prescriptions of topical benzyl benzoate dispensed in Belgian community pharmacies	National	NA	NA

#### Diagnoses of scabies

##### At general medical practices

Scabies diagnoses made by GPs were obtained from the INTEGO morbidity registry which provides publicly available data about disease incidence and prevalence aggregated by year, sex, and age group (https://www.intego.be/resultaten). The registry contains coded diagnoses extracted from electronic health records from over 100 general practice centers in Flanders, covering 6% of the Flemish population in 2023. In a given year, these data are based on the number of unique patients with a certain diagnosis, i.e., the numerator, with respect to total number of individuals with at least one GP visit in that year, i.e., the denominator, which is called the yearly contact group. Additional data is available upon request for researchers and policy makers [18]. We extracted the yearly number of scabies diagnoses per patient, per sex and age group for the period 2000–2023. Additionally, we requested the number of scabies diagnoses per quarter, per sex and age group and place of residence (LAU2 level) for the period 2011–2022. Incidences were calculated as the yearly or quarterly number of scabies diagnoses per 1,000 patients in the yearly contact group. As no quarterly denominators were available for the calculations of quarterly incidences, the respective yearly contact groups were divided by four.

##### At occupational health services

The external service for prevention and protection at work, IDEWE, is mainly active in the sectors of healthcare, education and government [19]. We obtained the yearly self-reported scabies diagnoses for which there was absenteeism or for which there was a link with work, per number of employees with at least one consultation at IDEWE during that year, from 2011 to 2022.

##### At refugee centers and outreach services for people in precarious living situations

We extracted the yearly number of scabies diagnoses in people sheltered in refugee centers from the Federal Agency for the Reception of Asylum Seekers (Fedasil), for 2016–2022 [20]. Scabies infestations were diagnosed upon admission (medical check-up) and during the person’s stay (medical consultation upon request of the patient). Incidences were calculated using the mean number of people sheltered in all refugee centers for the respective years as denominator.

In addition, we extracted data from the non-governmental organisation Doctors of the World, which ensures healthcare for people in precarious living situations (undocumented migrants and homeless people) who have no access to regular healthcare [21]. Medical consultations are performed by voluntary nurses and physicians, according to fixed schedules which are announced on site. We extracted the number of scabies diagnoses over the total number of consultations per year in the medical projects in Belgium, for the years 2019–2022, by age group, sex and month of consultation.

##### At student medical services in Leuven and Ghent

Yearly number of scabies diagnoses and number of consultations were obtained from student medical services in Leuven and Ghent, two student cities in Flanders, for the periods 2013–2022 and 2020–2022 respectively [22, 23].

#### Treatment reimbursements and sales data

National yearly sales data for permethrin (Zalvor^®^) for the period 2012–2022 were sourced from IQVIA (www.iqvia.com), which gathers information on pharmaceutical products purchased from wholesalers, covering 62% of Belgian pharmacies [24]. These data are then extrapolated by the data provider to reflect the total purchases made by community pharmacies. In addition, the National Institute for Health and Disability Insurance (NIHDI) provided monthly permethrin reimbursement data from November 2019 to March 2023, including information on age group, sex and province, as extracted from the Pharmanet database and covering approximately 99% of the Belgian population [25]. The Association of Pharmacists in Belgium (APB), provided monthly data on magistral preparations of benzyl benzoate sold in Belgian community pharmacies, from January 2019 till February 2023 [26]. No data on oral ivermectin were collected as it became available for scabies treatment only in 2022. Topical ivermectin is not often used and thus not included in our study. Data on general population growth was obtained from the Belgian Statistical Office.

### Data analysis

We described trends in scabies between 2000 and 2023. We conducted negative binomial regressions with scabies incidence or scabicide reimbursements or sales as the dependent variable and adjusting for time, sex and seasonality. We included an interaction term between year and sex to assess difference in trends between sexes. We calculated trends and their 95% confidence interval (95% CI). *P*-values for differences in proportions were calculated using Pearson’s chi-square tests. A *p*-value of 0.05 was considered for threshold of significance. All analyses were conducted in R version 4.2.1., using the MASS package for trend analysis [27, 28].

### Ethics approval

Surveillance of infectious diseases is a legal task of the service Epidemiology of Infectious Diseases from Sciensano, the Belgian Institute of Health. Ethical approval for the secondary use of the medical data collected by Doctors of the World for our study was granted by the Institutional Review Board of the Institute of Tropical Medicine in Antwerp, Belgium (Reference 1759/24).

## Results

### Diagnoses of scabies

#### At general medical practices

**Fig. 1:**
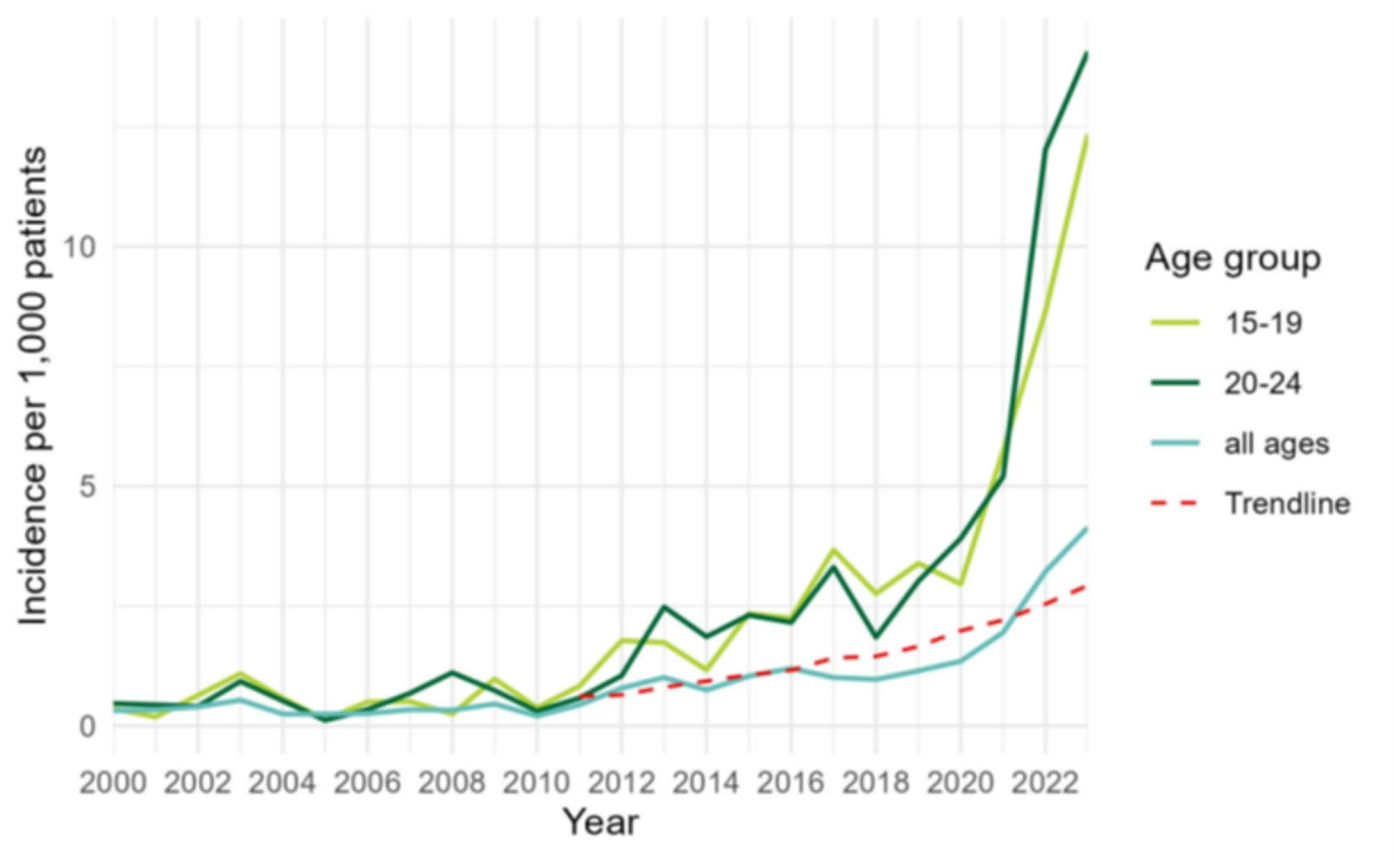
Incidence of scabies diagnoses in a GP network, all ages versus 15–19 and 20–24 years old, 2000–2023, with fitted trend since 2011 (start increase), Flanders (data source: INTEGO, Reference 1 in Table 1)

After a period with a stable incidence between 2000 and 2010, the yearly incidence of scabies diagnoses increased by almost 10 times between 2011 and 2023 (Fig. 1). This increase was particularly noticeable in 2021–2023. On average, the incidence increased by 15% annually between 2011 and 2023 (*p*-value < 0.001, 95% CI 12.9–17.9%) after adjusting for sex. The rise was more pronounced in younger age groups, specifically among those aged 15–24 years-old, reaching an incidence of 13.2/1,000 in 2023 compared to 4.1/1,000 for all ages (Fig. 1; Table 2). The incidence in men was 19% higher than in women (*p*-value 0.016, 95% CI 3.8–31.2%) but with similar trends (*p*-value 0.655) (data not shown). The colder months (October-March) had a 39% higher incidence compared to the warmer months (April-September) (*p*-value < 0.001, 95% CI 14.8–68.3%).

**Table 2:** Incidence of scabies diagnoses in a GP network by age group, 2019–2023, Flanders (data source: INTEGO, reference 1 in Table 1)

Age group (years)	Incidence per 1,000 patients
0–4	2.3
5–9	3.0
10–14	3.2
15–19	6.8
20–24	7.6
25–29	3.5
30–39	2.2
40–49	2.1
50–59	1.5
> 60	0.6

**Fig. 2:**
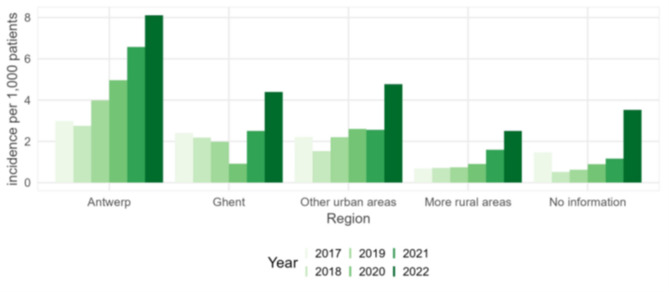
Incidence of scabies in a network of general practitioners, per place of residence of patients, 2017–2022, Flanders (data source: INTEGO, Reference 3 in Table 1)

From 2017 to 2022, the highest incidences in Flanders were reported in the largest city, Antwerp, and the lowest incidences in the more rural areas (Fig. 2). In 2022, the incidence in urban areas was 6.7/1,000 compared to 2.7/1,000 in rural areas. The increase in incidence over time was detected in all areas, independently of the rural/urban status. There were no differences in sex (*p*-value 0.412) or age distribution (*p*-value 0.783) between the different cities/areas.

#### At occupational health services

**Fig. 3:**
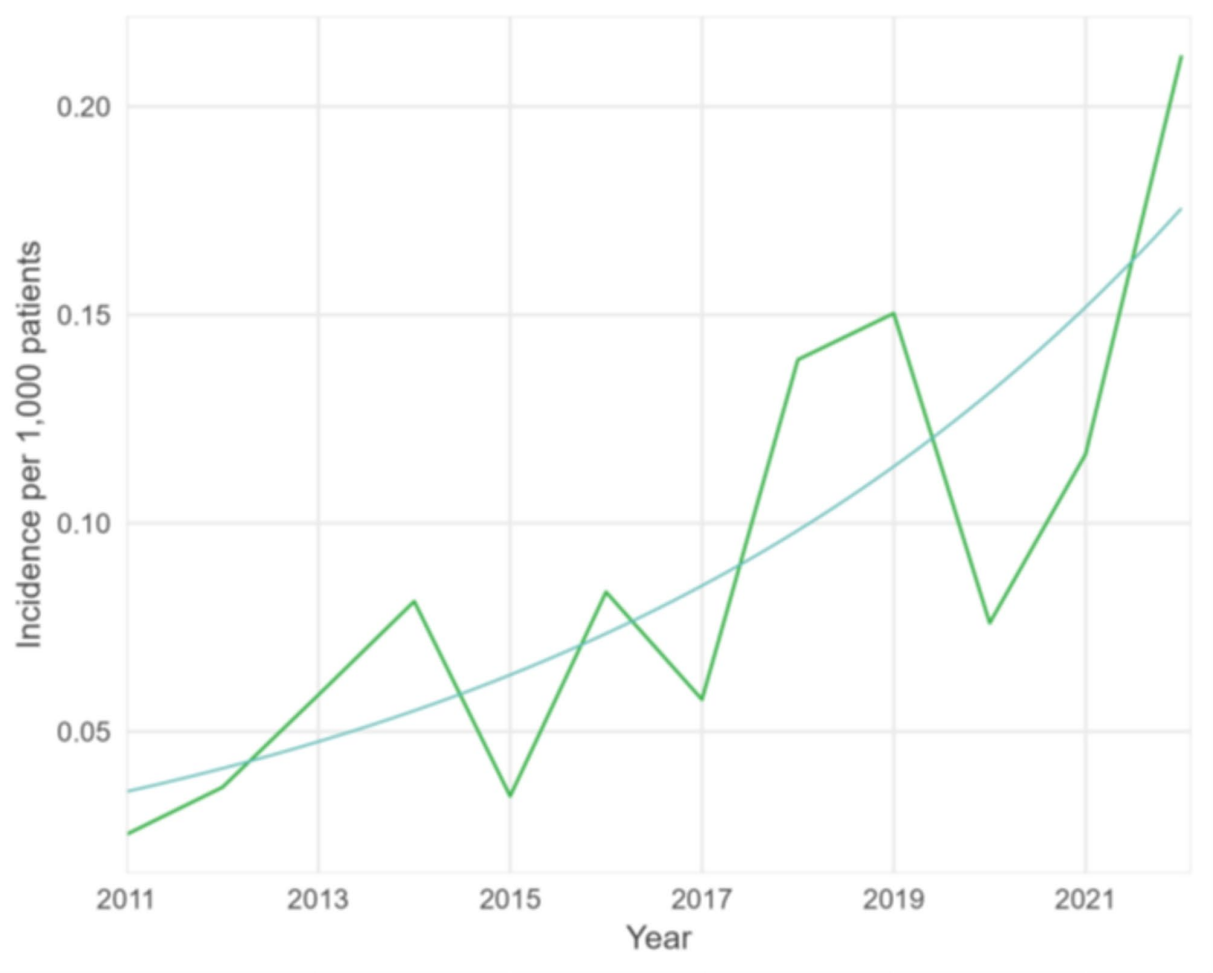
Number of scabies diagnoses per 1,000 employees with at least one consultation at IDEWE during that year, with fitted trend, 2011–2022, Belgium (data source: IDEWE, Reference 4 in Table 1)

Between 2011 and 2022, the number of registered scabies diagnoses per 1,000 employees who had at least one consultation at the occupational health services of IDEWE during that year, increased by 16% every year (*p*-value < 0.001, 95% CI 9.4–22.6%), with a temporary decrease in 2020–2021 (Fig. 3).

#### At refugee centers and outreach services for people in precarious living situations

**Fig. 4:**
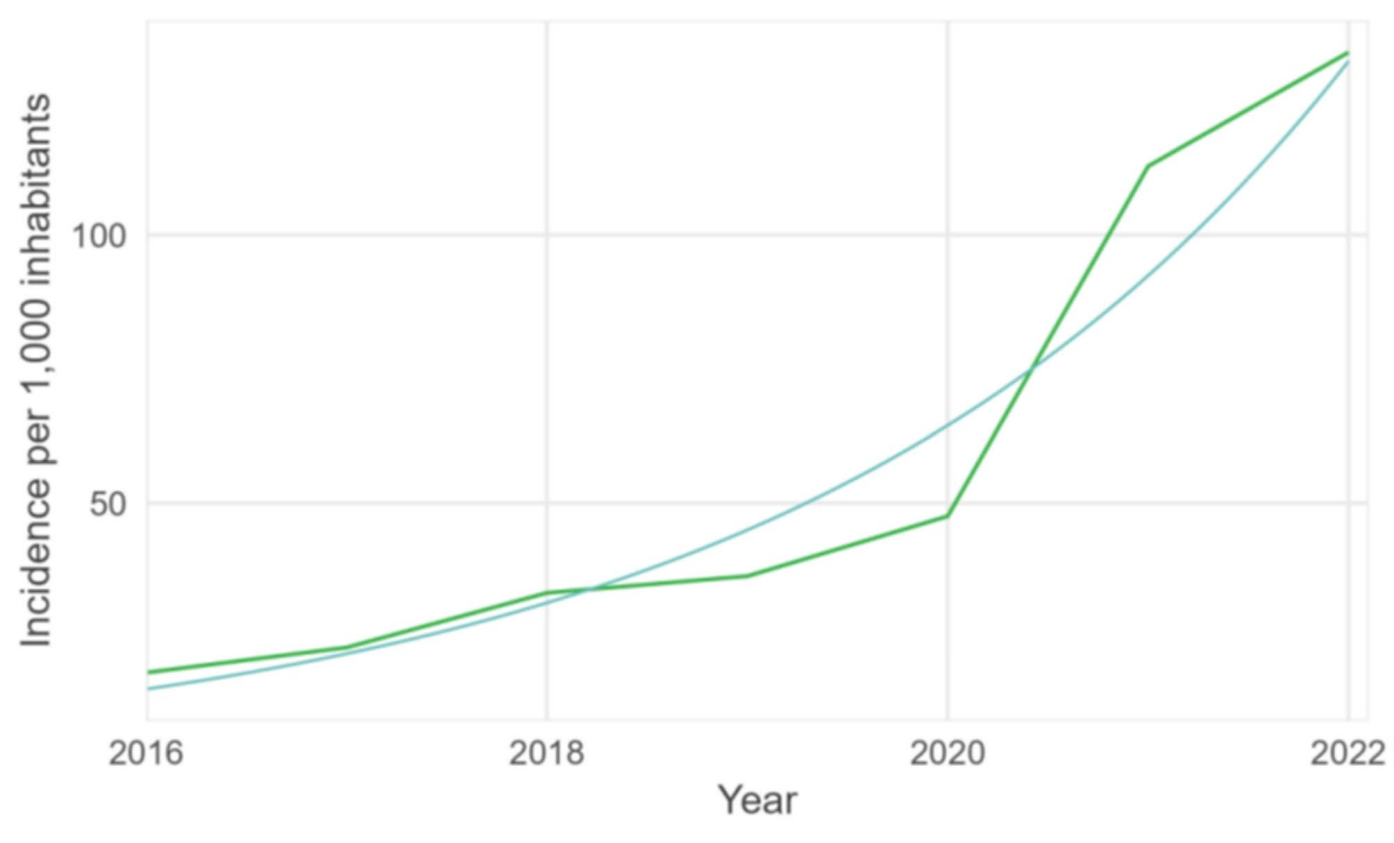
Yearly number of scabies cases per mean number of people sheltered in refugee centers of Fedasil with fitted trend, 2016–2022, Belgium (data source: Fedasil, Reference 5 in Table 1)

The yearly number of scabies diagnoses per mean number of persons sheltered in refugee centers from Fedasil increased by 41% every year between 2016 and 2022 (*p*-value < 0.001, 95% CI 32.5–49.9%) (Fig. 4). The increase was more pronounced in 2021 and 2022.

**Fig. 5:**
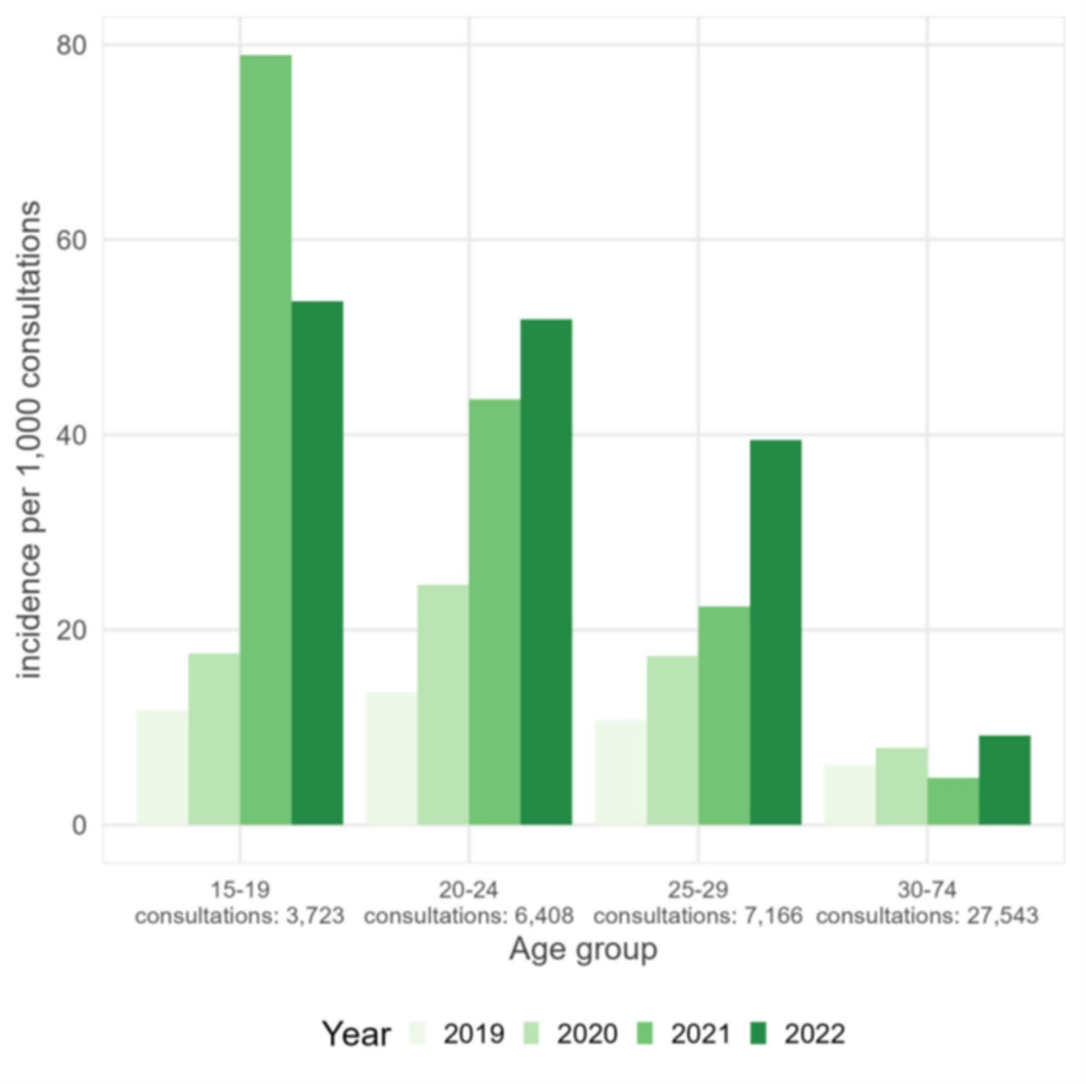
Yearly number of scabies diagnoses per 1,000 consultations per age group among patients of Doctors of the World, 2019–2022, Belgium. Age groups of 0–14 and 75 plus are left out because of very low numbers (data source: Doctors of the World, Reference 6 in Table 1)

From 2019 to 2022, the number of scabies diagnoses over the total number of consultations conducted at Doctors of the World increased by 41% every year (adjusted for sex, *p*-value < 0.001, 95% CI 32.1–50.0%). The incidence was highest in the age groups 15–19, 20–24 and 25–29 (Fig. 5; Table 3). The incidence was overall five times higher in males compared to females but with a similar increasing trend in males and females (*p*-value 0.202).

**Table 3:** Incidence of scabies per age group Doctors of the world, 2019–2022, Belgium (data source: Doctors of the world, reference 6 in Table 1)

Age group (years)	Number of scabies diagnoses per 1,000 consultations
0–4	2.3
5–9	3.0
10–14	3.2
15–19	6.8
20–24	7.6
25–29	3.5
30–39	2.2
40–49	2.1
50–59	1.5
> 60	0.6

#### At student medical services in Leuven and Ghent

The incidence of scabies diagnoses at student medical services increased in both Leuven and Ghent. In Leuven, the incidence rose from 1.09 scabies diagnoses per 1,000 consultations in 2013 to 5.72 in 2023, and in Ghent from 6.00 per 1,000 consultations in 2020 to 10.40 in 2022.

### Treatment reimbursements and sales data

**Fig. 6:**
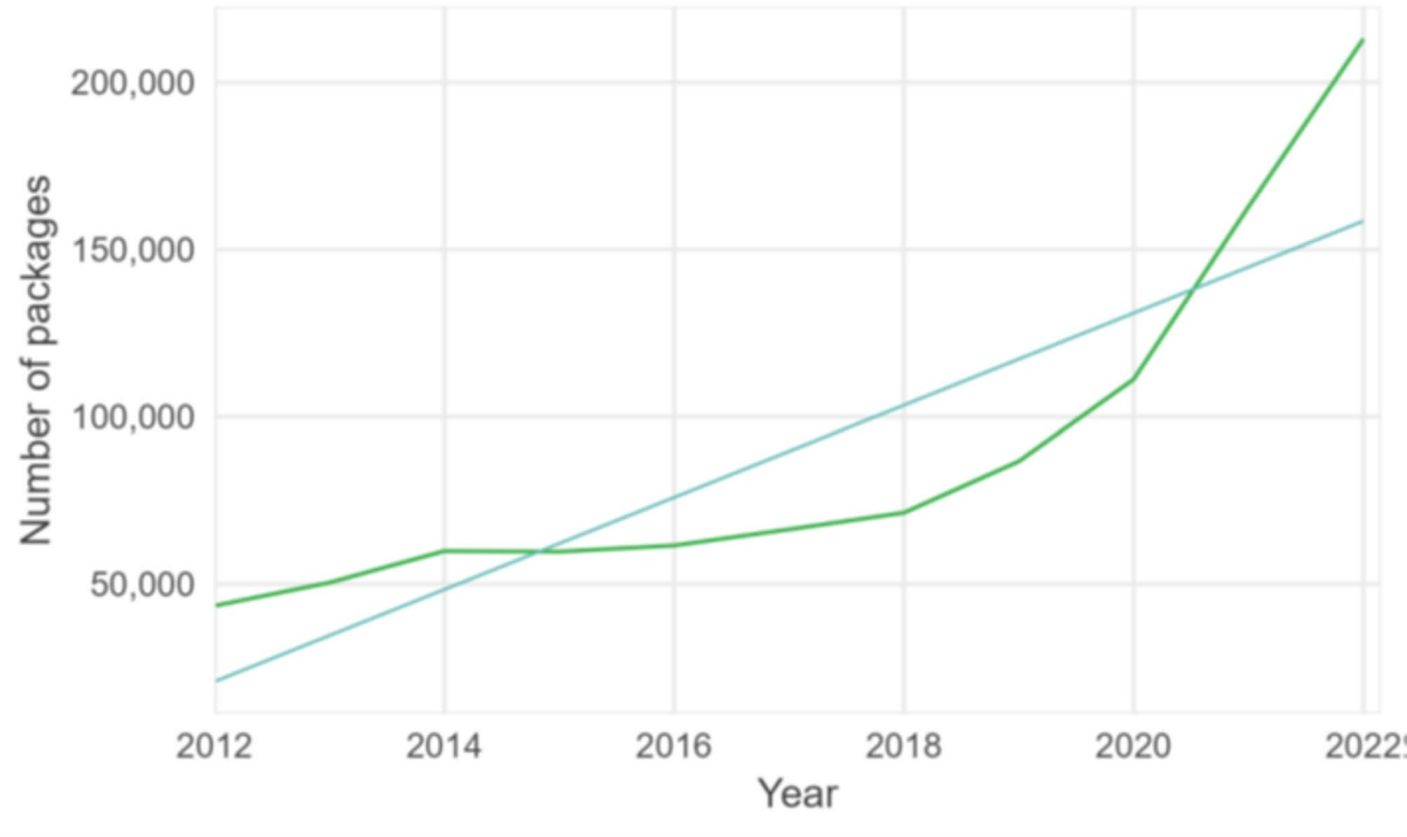
Number of packages of permethrin sold in community pharmacies with fitted trend, 2012–2022, Belgium (data source: IQVIA, Reference 9 in Table 1)

Reimbursements and sales of scabies treatments also increased over time. The number of permethrin packages bought by community pharmacies from wholesalers quadrupled between 2012 and 2022, with an average yearly increase of 15% (*p*-value < 0.001, 95% CI 12.0-18.6%) (Fig. 6). In this period, the Belgian population increased by 0.2 to 0.6% per year [29].

**Fig. 7:**
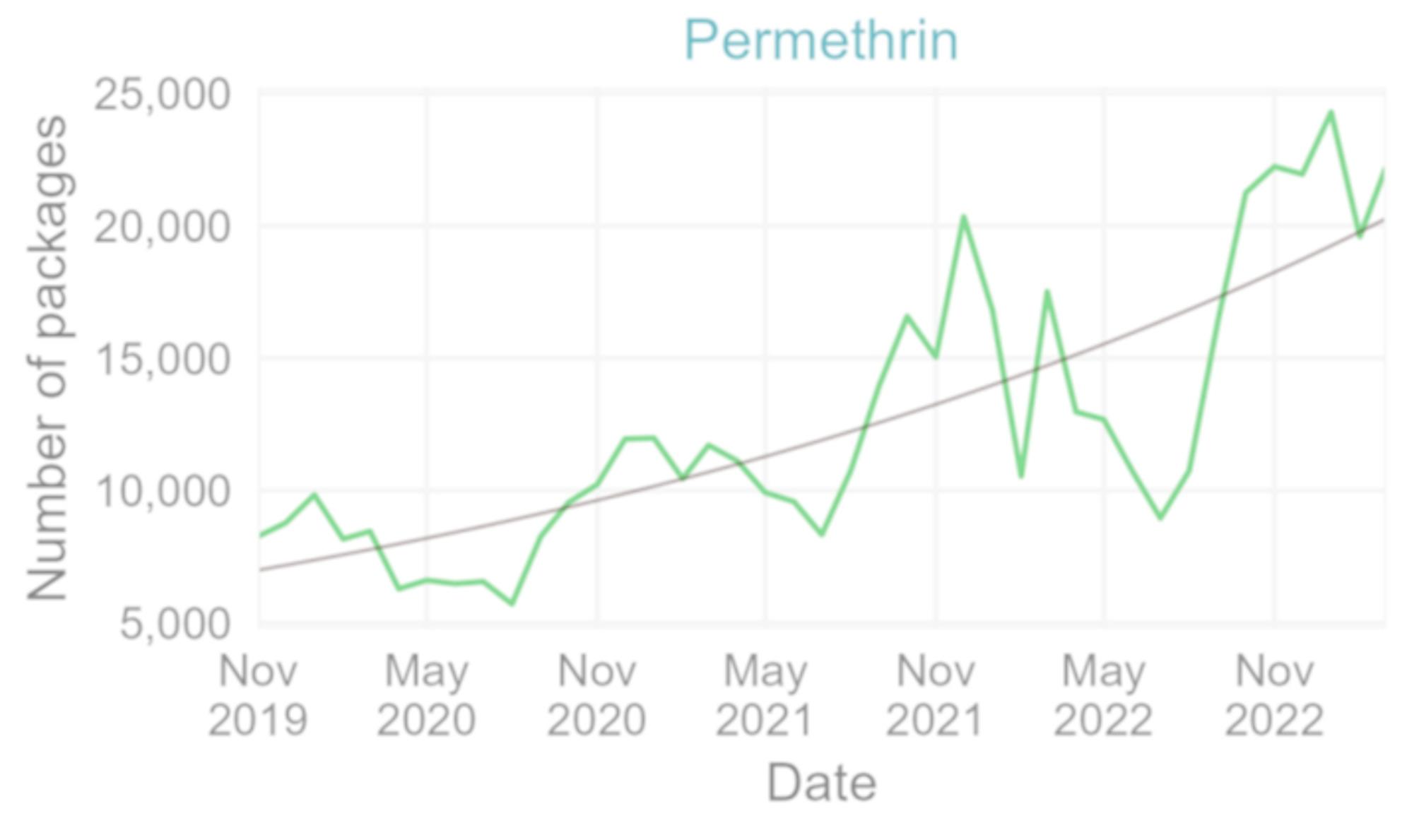
Number of reimbursed permethrin packages per month November 2019– March 2023 with fitted trend, Belgium (data source: NIHDI, Reference 10 in Table 1)

**Fig. 8:**
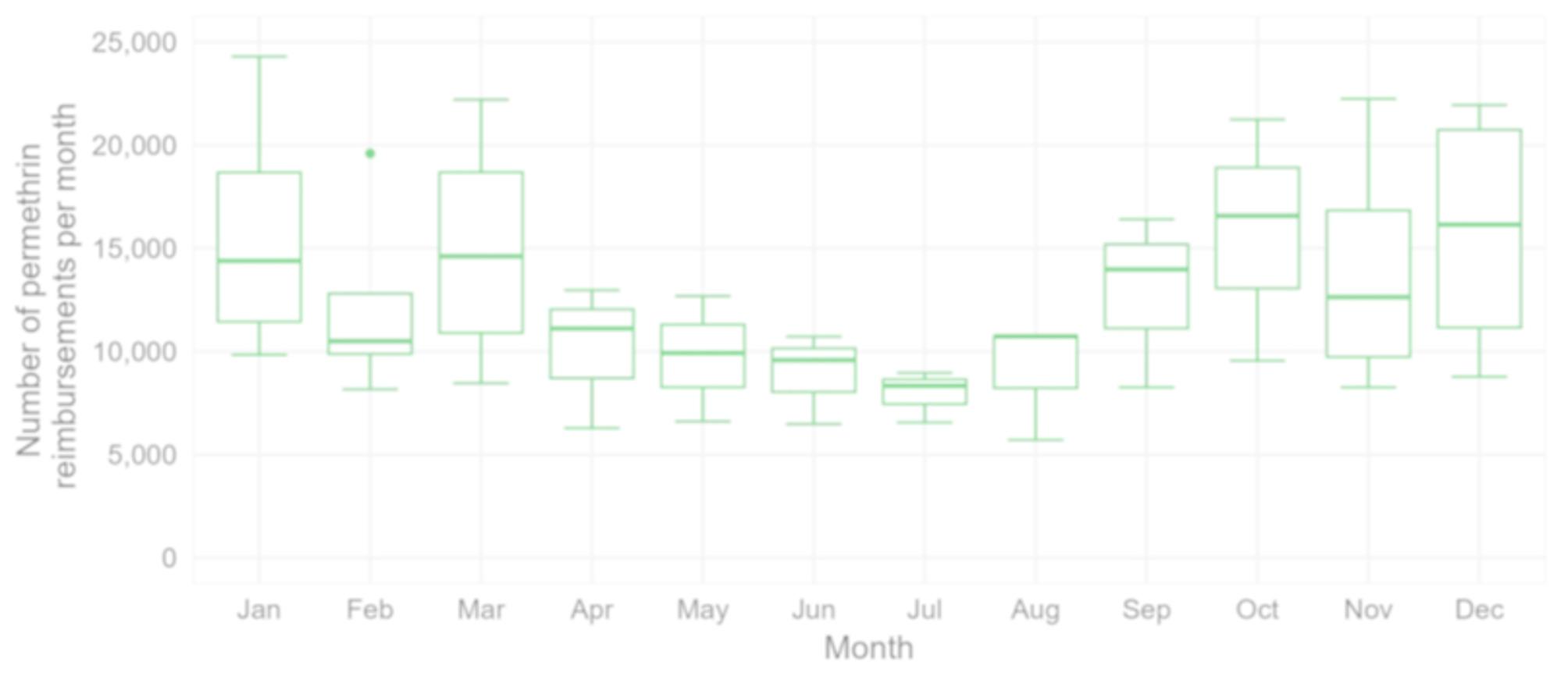
Boxplot of the number of reimbursed permethrin packages per month, November 2019 - March 2023, Belgium (horizontal lines denote lower quartile, median and upper quartile, with dots showing outliers) (data source: NIHDI, Reference 10 in Table 1)

The number of reimbursed packages of permethrin increased on average by 3% every month between November 2019 (start of reimbursement) and March 2023 (*p*-value < 0.001, 95% CI 2.1–3.3%) (Fig. 7), with higher numbers in the winter months (Fig. 8).

There was no significant difference in number of reimbursed packages nor in trend between females and males (*p*-value 0.376). 60% (60%) of the reimbursements for permethrin were for those aged 20–59 years (Table 4). Brussels showed a slower increase compared to Flanders (*p*-value < 0.001) and Wallonia (*p*-value < 0.001) but the general trend and seasonality were similar across the three regions of Belgium. Of the permethrin reimbursements, 18% occurred for patients residing in the province of Liège, whereas this province contains only 10% of the Belgian population (Table 4) [29].

**Table 4:** Permethrin packages reimbursed, November 2019-March 2023, belgium. Percentage per characteristic subgroup and comparison with Belgian population 2022 (data source: NIHDI, reference 10 in Table 1)

Characteristic		Number (%) of packages reimbursed per characteristic subgroup (Total = 513,840)	Proportion of the characteristic subgroup in the Belgian population
Sex	Female	266,580 (52%)	51%
	Male	246,716 (48%)	49%
	NA	544	NA
Age group	0–19 years old	155,045 (30%)	22%
	20–59 years old	303,621 (60%)	52%
	≥ 60 years old	49,816 (10%)	26%
	NA	5,358	NA
Region/province	Brussels	41,646 (8%)	10%
	Flanders		58%
	Antwerp	93,176 (18%)	16%
	East Flanders	50,312 (10%)	13%
	Flemish Brabant	35,671 (7%)	10%
	Limburg	25,287 (5%)	8%
	West Flanders	45,873 (9%)	11%
	Wallonia		32%
	Hainaut	68,818 (13%)	12%
	Liège	93,795 (18%)	10%
	Luxemburg	13,206 (3%)	2%
	Namur	33,080 (7%)	4%
	Walloon Brabant	10,392 (2%)	4%
	NA	2,584	NA
Year	2019*	17,041	-
	2020	98,122	-
	2021	149,791	-
	2022	182,792	-
	2023^†^	66,094	-

*November-December, †January-March, NA = not available

**Fig. 9:**
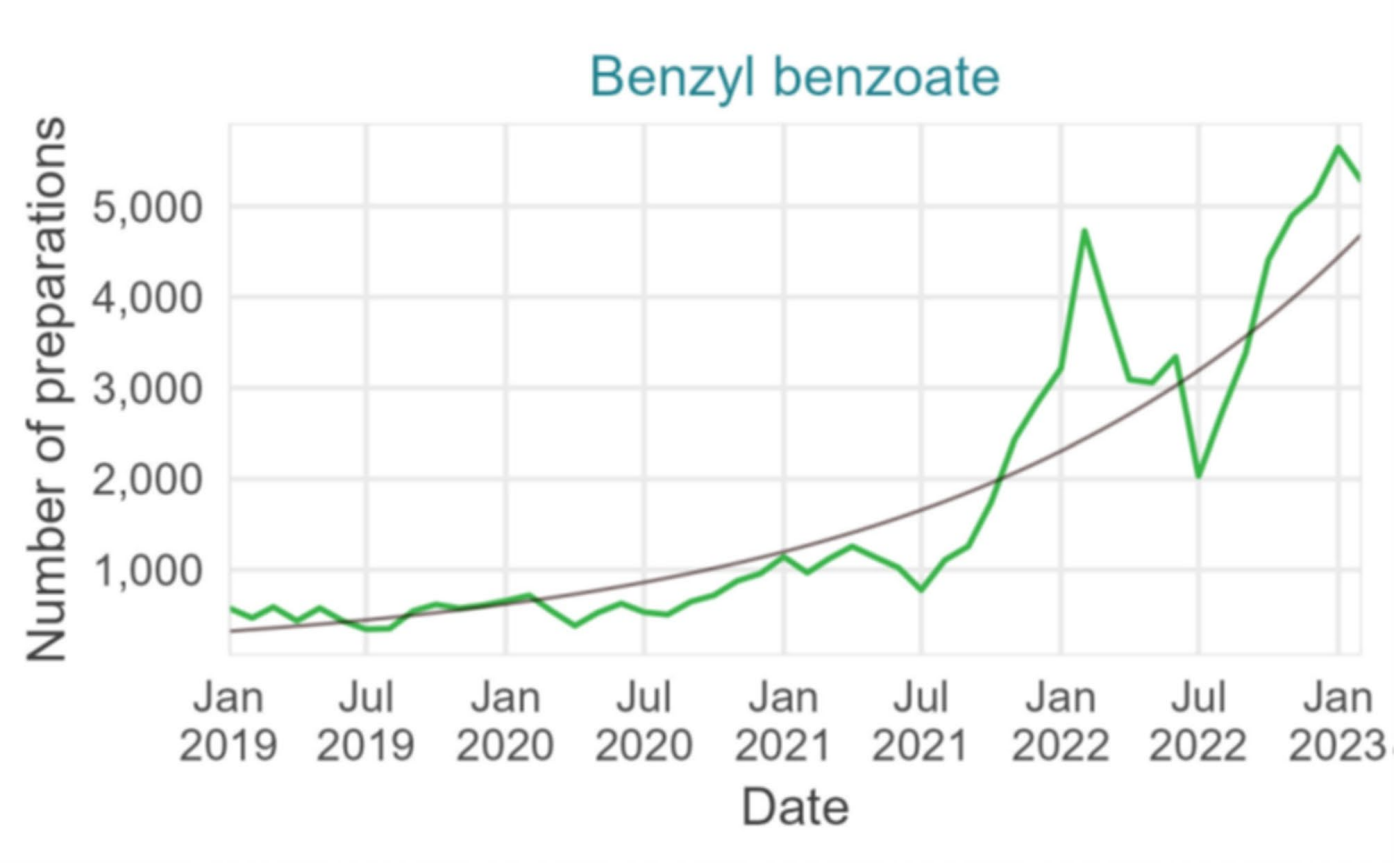
Number of benzyl benzoate preparations dispensed in pharmacies per month with fitted trend, January 2019– February 2023, Belgium (data source: Algemene Pharmaceutische Bond, Reference 11 in Table 1)

There was a 7-times increase in benzyl benzoate magistral preparations dispensed between 2019 and 2022 with an average monthly increase of 6% (*p*-value < 0.001, 95% CI 5.0-6.2%) (Fig. 9).

## Discussion

To our knowledge, this is the first description of the epidemiology of scabies in Belgium over the past two decades [30]. All investigated data sources showed an increasing trend with a notably high incidence in 2021–2023. According to the GP network INTEGO, this increase started in 2011, following a period with a stable incidence. Although this network is active only in one of the three Belgian regions, the same increasing trend was observed in the national data on treatment sales and reimbursements, with no significant regional difference. We thus consider the results from the GP network in Flanders reflective of the trends in the entire country.

It is unlikely that the increase in cases was due to a change in diagnostic guidelines, as the guidelines from RHAs were only amended after the period covered by this study, in an attempt to halt the increase. The increase in the number of reimbursed packages of permethrin is not solely linked to the start of the reimbursement in November 2019, as an increase was already evident in yearly sales data before 2019. Moreover, prescriptions of benzyl benzoate showed a similar increasing trend during the same period, further confirming an actual increase in scabies diagnoses.

Clinicians consider that increasing resistance to permethrin and ivermectin, poor compliance with complex hygiene measures, and a lack of clear communication to patients are potential drivers of the surge. Both in vitro studies and case reports suggest increasing resistance to permethrin and ivermectin [31–33]. Studies on potential resistance of the *Sarcoptes* mite to scabies treatments are however challenging to conduct [34] and successful treatment is hard to establish with certainty. Additionally, treatment failure can be linked to numerous factors, such as incorrect application of topical treatment or suboptimal compliance with treatment or hygienic measures. There have been periods of temporary limited availability of permethrin at pharmacies in Belgium during the study period. This might have exacerbated the spread of scabies, due to interrupted treatments, despite benzyl benzoate still being available as a second-line treatment. Before the start of reimbursement in November 2019, which reduced the price of one permethrin treatment from 15.35€ to 3.37€ for the patient, the cost of permethrin for treating cases and their household contacts might have prevented economically disadvantaged patients to access treatment [35]. The recent availability of oral ivermectin for scabies treatment, could enhance successful treatment in patients for whom application of topical treatments is challenging, such as people with reduced mobility or living in collectivities. However, until June 2025, oral ivermectin was not reimbursed, and the cost for the patient remained a disadvantage (28,01€ commercial package of four tablets, approximately 8€ four tablets magistral preparation) [36].

The incidence we observed was higher in males compared to females but the trend was similar between the two. Findings in other studies vary as some reported higher incidences in females, while more recent studies described a higher incidence in males [5, 6, 37]. A German study attributed higher incidence in males to stronger social connectivity, especially among male adolescents and young adults [5]. Among people in precarious living situations, we registered five times higher incidences in males compared to females. Male asylum seekers arriving in Belgium without family have more difficulties in finding shelter and are more likely to sleep on the street in close contact groups. This may make them more vulnerable to scabies transmission.

The increase detected in our study was more pronounced in adolescents and young adults (15 to 29 years old), consistent with findings from other Western European countries [5, 6, 9]. These higher incidences in young age groups might be linked to more alternating close contacts and lower adherence to therapy and hygiene measures [5, 38]. Our study did not find high incidences in children younger than 15.

In Flanders, cities generally had higher incidences compared to rural areas. People living in larger cities might have a broader and more diverse social network compared to more rural areas, contributing to increased transmission rates. Additionally, in Belgium, more people living in cities are at risk of poverty compared to those living in rural areas, and people with lower income have more unmet needs for medical care [39, 40]. A German study on Internet search engine data related to scabies, observed a positive correlation between the average searches and population density [41]. The number of reimbursed permethrin packages was higher in the province of Liège, Wallonia, compared to other provinces, accounting for population size. Discussions with the RHAs highlighted the presence of a readily accessible dermatology ward in the area which could partially explain this higher use of scabies treatment, as more cases are diagnosed.

The increase in incidence recorded in refugee centers of Fedasil, with highest incidences in 2021–2022, does not seem to be driven by higher shelter occupancy which could have led to higher transmission rates. The occupancy in 2016 was similar to that in 2021 and 2022, yet scabies incidence was seven times lower in 2016 compared to 2021–2022. A study in Greece reported scabies as the third most frequent infectious disease among asylum seekers, with an increasing trend [42]. The authors of this Greek study refer to conditions on the transnational migrating routes and rapid turnover at the refugee centers as potential drivers for the high burden of scabies among asylum seekers.

Both data from the GP network in Flanders and the national monthly number of reimbursements of permethrin indicate a seasonal trend, with more scabies infestations in autumn and winter. Similar trends have been observed in other European countries [6–9]. Transmission rates are probably higher when people spend more time together indoors (physical crowding) and cold environments increase the survival time of scabies mites [43].

The temporary decrease in scabies diagnoses registered by IDEWE in 2020 can likely be linked to the start of the COVID-19 pandemic. Lockdowns and telework led to fewer consultations at occupational health services during those first months. Other data sources did not show an overall decrease of scabies in 2020, unlike what was seen for other infectious diseases. We even noticed a sharper increase since 2020 especially in the adolescents and young adults. This is similar to reports from the Netherlands, where some social distancing measures like curfews may have had the opposite effect among this age group, leading to more sleep-overs [9]. So far, we do not have a clear explanation as to why the incidence in Ghent decreased in 2020 whereas this was not the case for the other areas in Flanders.

The results of this study should be interpreted in light of a number of limitations. We combined different data sources, not primarily intended for surveillance and not uniform across all regions of Belgium. However, because all consulted data sources indicate the same trend, we are confident our study confirms the increase even if we are not able to quantify the actual number of scabies infestations in each part of the Belgian population. The data from services offered to various population groups originate from different sources, each employing distinct data collection methods and denominators. Consequently, this study is not suited for comparisons between these groups or for drawing inferences about the factors or individuals contributing to the scabies epidemic in Belgium. We also did not have access to data on scabies diagnoses by dermatologists. This was alleviated by analyzing sales and reimbursements data. There were clusters reported from the RHAs but these were not included in the manuscript due to recent implementation of the reporting. Finally, from the available data sources, we could not determine what proportion of cases or treatment sales were linked to treatment failures, reinfestations, new infestations, or treatment of close contacts.

The number of cases registered in the GP network are probably an underestimation, as scabies is still largely underdiagnosed. Children for instance can present with atypical lesions and scabies is often misdiagnosed as eczema and GPs do not have access to confirmation tools. New diagnostic guidelines by the RHA in Flanders were published in 2025 to improve case ascertainment. We also recommend training support to GPs on diagnosis and contact tracing. Our team is now conducting studies with both GPs and dermatologists to better understand the real incidence, driving factors for the increase as well as their challenges in the management of scabies.

## Conclusions

In conclusion, based on the available data sources there is a clear increasing trend of scabies in Belgium. It is crucial to conduct studies to better quantify the increase and to investigate the proportion of treatment failures and new infestations, to disentangle the reasons for treatment failures, and to understand other underlying drivers of the increase. A standardized follow-up of the epidemiology with data sources uniform over the entire country and stable over time is urgently needed. To that end, the ICPC2 code for scabies was recently added to a sentinel GP surveillance tool of the National Institute of Health, and data on the use of permethrin and ivermectin will be monitored.

## Data Availability

The datasets used and/or analysed during the current study are available from the corresponding author on reasonable request.

## Abbreviations

%: Percent
€: Euros
APB: Association of Pharmacists in Belgium
BAPCOC: Belgian Antibiotic Policy Coordination Commission
CI: Confidence Interval
e.g.: For example
FEDASIL: Federal Agency for the Reception of Asylum Seekers
GP: General Practitioner
ICPC: International Classification of Primary Care
LAU: Local Administrative Unit
NIHDI: National Institute for Health and Disability Insurance
RHA: Regional Health Authority
